# HIV care in Yangon, Myanmar; successes, challenges and implications for policy

**DOI:** 10.1186/s12981-017-0137-z

**Published:** 2017-03-04

**Authors:** Ne Myo Aung, Josh Hanson, Tint Tint Kyi, Zaw Win Htet, David A. Cooper, Mark A. Boyd, Mar Mar Kyi, Htin Aung Saw

**Affiliations:** 1 Department of Medicine, University of Medicine 2, Yangon, Myanmar; 2 Menzies School of Health Research, Darwin, Australia; 3 The Kirby Institute, Sydney, Australia; 4 Department of Medical Care, Ministry of Health, Nay Pyi Taw, Myanmar; 5 Insein General Hospital, Yangon, Myanmar; 6 University of Adelaide, Adelaide, Australia

**Keywords:** HIV, Myanmar, Anti-retroviral therapy, Tuberculosis, Isoniazid prophylaxis therapy, Key affected populations, Resource limited settings

## Abstract

**Background:**

Approximately 0.8% of adults aged 18–49 in Myanmar are seropositive for Human Immunodeficiency Virus (HIV). Identifying the demographic, epidemiological and clinical characteristics of people living with HIV (PLHIV) is essential to inform optimal management strategies in this resource-limited country.

**Methods:**

To create a “snapshot” of the PLHIV seeking anti-retroviral therapy (ART) in Myanmar, data were collected from the registration cards of all patients who had been prescribed ART at two large referral hospitals in Yangon, prior to March 18, 2016.

**Results and discussion:**

Anti-retroviral therapy had been prescribed to 2643 patients at the two hospitals. The patients’ median [interquartile range (IQR)] age was 37 (31–44) years; 1494 (57%) were male. At registration, injecting drug use was reported in 22 (0.8%), male-to-male sexual contact in eleven (0.4%) and female sex work in eleven (0.4%), suggesting that patients under-report these risk behaviours, that health care workers are uncomfortable enquiring about them or that the two hospitals are under-servicing these populations. All three explanations appear likely. Most patients were symptomatic at registration with 2027 (77%) presenting with WHO stage 3 or 4 disease. In the 2442 patients with a CD4+ T cell count recorded at registration, the median (IQR) count was 169 (59–328) cells/mm^3^. After a median (IQR) duration of 359 (185–540) days of ART, 151 (5.7%) patients had died, 111 (4.2%) patients had been lost to follow-up, while 2381 were alive on ART. Tuberculosis (TB) co-infection was common: 1083 (41%) were already on anti-TB treatment at registration, while a further 41 (1.7%) required anti-TB treatment during follow-up. Only 21 (0.8%) patients were prescribed isoniazid prophylaxis therapy (IPT); one of these was lost to follow-up, but none of the remaining 20 patients died or required anti-TB treatment during a median (IQR) follow-up of 275 (235–293) days.

**Conclusions:**

People living with HIV in Yangon, Myanmar are generally presenting late in their disease course, increasing their risk of death, disease and transmitting the virus. A centralised model of ART prescription struggles to deliver care to the key affected populations. TB co-infection is very common in Myanmar, but despite the proven efficacy of IPT, it is frequently not prescribed.

## Background

Myanmar is a country at a critical point in its history. After five decades of military rule, the newly elected government faces enormous challenges in improving the health, welfare and prosperity of its people [1, 2]. The human immunodeficiency virus (HIV) epidemic is one of the country’s greatest health concerns: in 2015, 0.8% of the country’s adults aged 18–49 were seropositive [3]. Myanmar’s health system remains poorly resourced and under-equipped to achieve the UNAIDS “90–90–90” target or the ambitious Sustainable Development Goal of ending the AIDS epidemic by 2030 [4, 5]. HIV prevalence is highest in people who inject drugs (PWID), men who have sex with men (MSM) and female sex workers (FSW) [6]. However, it is frequently challenging for Myanmar health services to reach these key affected populations (KAPs) and ensure that they are receiving optimal care [6].

One of the key aspects of the country’s response to the HIV epidemic has been a rapid scale up of anti-retroviral therapy (ART). In 2015, 111,563 individuals—representing an estimated 50% of the people living with HIV (PLHIV)—were receiving ART and the rollout has accelerated since [6]. All PLHIV in Myanmar receiving ART have basic demographic, epidemiological and clinical data collected prospectively and entered into the National AIDS Programme’s “Patient HIV Care and Antiretroviral Treatment (ART) Record”, commonly referred to as “The White Card”. The White Card records a patient’s gender, age, address and marital and employment status. It documents their referral origin, their risk factors for infection, the CD4+ count, the WHO disease stage and the presence or history of tuberculosis. It also records the prescribed ART regimen and if a patient dies or is lost to follow up.

These simple data provide a useful insight into the characteristics of the PLHIV receiving ART, and therefore might inform approaches for the optimal delivery of care. Just as importantly, the absence of key populations in these data might highlight PLHIV who are not receiving ART, permitting policy makers to develop strategies to reach under-serviced populations, improving the health of these often vulnerable populations, and also preventing ongoing transmission [6–8].

This study examined White Card data collected at two large ART prescribing centres to create a “snapshot” of the HIV epidemic in Myanmar. It was hoped that this might both identify the successes of the current programme and the challenges that remain.

## Methods

The White Cards of all PLHIV commencing ART between the 6th June 2001 and the 18th March 2016 at two Yangon hospitals—Waibargi Hospital (a specialist infectious disease hospital) and Insein General Hospital (a tertiary referral general hospital)—were reviewed. These data were de-identified and transferred from the hospital databases into an electronic database (Microsoft Excel); statistical analysis was performed using statistical software (Stata, version 10). Death was either witnessed in hospital or reported by the next of kin. If there was no contact for 3 months after the last outpatient visit, the patient was defined as lost to follow up. Groups were analysed using the Kruskal–Wallis and Chi squared tests. Multivariate analysis was performed using backwards stepwise logistic regression, with only variables that were significant in univariate analysis at a significance level of <0.05 selected for multivariate analysis. Ethical approval for the study was granted by the Human Research Ethics Committees of the University of Medicine 2, Yangon and the Menzies School of Health Research, Darwin, Australia. The Committees waived the requirement for informed consent as the publication of retrospective, aggregated, de-identified data was felt to pose negligible risk to the participants.

## Results

There were 2643 patients with a completed white card at the two hospitals, 1674 at Waibargi Hospital and 969 at Insein General Hospital; their characteristics are presented in Tables 1 and 2. The median [interquartile range (IQR)] age of the patients was 37 (31–44) years; 1494 (57%) patients were male. Most patients were referred after having presented with symptoms to the outpatient department (1959 patients, 74%) or after an inpatient admission (449 patients, 17%); 68 patients (2.6%) presented for voluntary testing while only two patients (0.1%) were referred from a drug treatment service.

**Table 1:** Characteristics of the patients at the two study sites

Variable	All n = 2643	Waibargi Hospital n = 1674	Insein Hospital n = 969	p^
Age	37 (31–44)	38 (32–45)	35 (29–41)	<0.001
Gender (% male)	1494 (57%)	1011 (60%)	483 (50%)	<0.001
Unemployed	917 (35%)	453 (27%)	464 (48%)	<0.001
Living in the same region^a^	2042 (80%)	1117 (70%)	925 (95%)	<0.001
CD4+ at registration	169 (59–328)	159 (50–332)	185 (74–323)	0.03
WHO stage at registration	3 (3–3)	3 (3–4)	3 (1–3)	<0.001
Prescribed TDF + FTC + EFV	2280 (86%)	1420 (85%)	860 (89%)	0.005
CD4+ change after ART (cells/mm^3^)	148 (46–263)	141 (44–258)	166 (52–276)	0.19
Required anti-tuberculosis treatment	1124 (43%)	691 (41%)	433 (45%)	0.09
Isoniazid prophylaxis therapy	21 (0.8%)	0	21 (2%)	<0.001
Co-trimoxazole prophylaxis therapy	2125 (81%)	1254 (76%)	871 (90%)	<0.001
Died	151 (5.7%)	116 (7.3%)	35 (3.7%)	<0.001
Lost to follow up	111 (4.2%)	89 (5.3%)	22 (2.3%)	<0.001

All numbers represent median and interquartile range or absolute number (percentage)

*WHO* World Health Organization, *TDF* tenofovir disoproxil fumarate, *FTC* emtricitabine, *EFV* efavirenz, *ART* anti-retroviral therapy

^ Comparison between the study sites using Kruskal–Wallis or Chi squared test

^a^75 patients did not have an address recorded; all were at Waibargi Hospital

**Table 2:** Comparison between patients who had died or who were alive on ART at the end of the study period

Variable	All n = 2643	Died n = 151^a^	Alive on ART n = 2381^a^	p^
Age	37 (31–44)	40 (33–45)	37 (31–44)	0.001
Gender (% male)	1494 (57%)	96 (64%)	1327 (56%)	0.06
Unemployed	917 (35%)	64 (42%)	814 (34%)	0.04
Living in a different region^b^	526 (20%)	43 (30%)	462 (20%)	0.005
CD4 + count at registration (cells/mm^3^)	169 (59–328)	45 (23–127)	183 (65–337)	<0.001
WHO stage at registration	3 (3–3)	4 (3–4)	3 (2–3)	0.0001
On treatment for tuberculosis	1124 (43%)	99 (66%)	971 (41%)	<0.001
Isoniazid prophylaxis therapy^c^	21 (0.8%)	0	20 (0.8%)	0.26
Co-trimoxazole prophylaxis therapy^d^	2125 (81%)	136 (93%)	1904 (80%)	<0.001

All numbers represent median and interquartile range or absolute number (percentage)

^ Comparison between survivors and those that died using Kruskal–Wallis or Chi squared test

^a^Does not include the 111 patients in the cohort lost to follow up

^b^75 patients did not have an address recorded; 6 patients who died and 69 still alive

^c^One patient lost to follow up

^d^2627 (99%) had data regarding co-trimoxazole prescription

All patients had data collected on risk factors for infection: 2271 (86%) were recorded as having acquired the infection heterosexually, 82 (3.1%) were recorded as having acquired the infection from a blood transfusion. Only 22 (0.8%) were documented to have acquired the infection through injecting drug use, while male-to-male sexual contact was reported in eleven (0.4%) and eleven (0.4%) reported that they were female sex workers. Of the 2643 patients, 1545 (58%) were married; 664 (43%) had an HIV positive spouse, while in 207 (13%) cases the spouse was negative and in 674 (44%) the HIV status of the spouse was unknown. Waibargi Hospital looked after a greater number of patients from outside the Yangon region: 30% versus 4.5% at Insein General Hospital (p < 0.001).

Patients generally presented late in the course of the disease: 2027 (77%) presented with WHO stage 3 or 4 disease. In the 2442 patients with a CD4+ T-cell count recorded at registration, the median (IQR) CD4+ count was 169 (59–328) cells/mm^3^. Tuberculosis was common in the cohort: 1083 (41%) were already on treatment for tuberculosis at the time of registration, while a further 41 (1.7%) required treatment for tuberculosis after registration. Despite the high rate of tuberculosis, only 21 patients in the cohort were prescribed isoniazid prophylaxis therapy (IPT) during the study period. There were 2627 patients with data available regarding co-trimoxazole prophylaxis (CPT), 2125 (81%) of whom received this therapy during the study period, including 1158 (85%) of the 1358 patients with a CD4+ count of <200 cells/mm^3^.

In the 2450 patients for whom the exact date of ART commencement was available, it was initiated at a median (IQR) of 21 (9–42) days after the HIV diagnosis was recorded. One hundred and seven (4.4%) PLHIV started ART on the same day that the diagnosis was recorded. The most commonly prescribed antiretroviral (ART) regimen was tenofovir, emtricitabine and efavirenz, with 2280 (86%) receiving this combination. In the 1247 patients with a recorded follow up CD4+ T-cell count at least 90 days after registration, there was a median increase in the CD4 count of 150 (47–263) cells/mm^3^; 192 (15.1%) had a fall in their CD4+ count.

At the time of data analysis, a median (IQR) of 359 (185–540) days after patient registration, 151 (5.7%) patients had died, 111 (4.2%) had been lost to follow up and 2381 (90.1%) were alive and taking ART (Table 1). In multivariate analysis, the independent predictors of death were a CD4 + count at registration of <200 cells/mm^3^ (odds ratio (OR) 6.4, 95% confidence interval (CI) 3.5–11.6, p < 0.0001); treatment at Waibargi Hospital (OR (95% CI) 1.9 (1.2–2.8), p = 0.004); unemployment (OR (95% CI) 1.8 (1.3–2.7) p = 0.002); age >40 years (OR (95% CI) 1.6 (1.1–2.4) p = 0.008) and a requirement for TB therapy (OR (95% CI) 1.6 (1.1–2.4) p = 0.02). The only variable associated with loss to follow up in either univariate or multivariate analysis was treatment at Waibargi Hospital (OR (95% CI) 2.4 (1.5–3.9), p < 0.001). All 21 patients receiving IPT were being treated at IGH. Of these 21, one was subsequently lost to follow up but none of the remaining 20 died or required TB treatment during the follow up period.

## Discussion

People living with human immunodeficiency virus at these two large treatment centres in Myanmar are presenting late in their disease course. The resulting delay in initiating ART increases their risk of disease and death and the likelihood of transmitting the virus [8, 9]. Tuberculosis co-infection is very common, but despite the proven efficacy of IPT [10]—and a strong recommendation for its prescription in WHO guidelines [11]—it was prescribed rarely in this cohort. The greatest burden of HIV in Myanmar is among PWID, MSM and FSW [6], however, very few patients in this cohort reported these risk behaviours at registration. This suggests that either these large ART prescribing centres are not servicing these KAPs, patients avoid acknowledging these risk behaviours or that health care workers are uncomfortable enquiring about them. All three explanations appear likely.

The late presentation of Myanmar PLHIV in this series resembles the findings in studies in other resource-limited settings [12–16]. Late presentation is not restricted to patients with HIV infection in a country whose public health care system is critically under-resourced [2]. In a 2012 WHO report, Myanmar was the country with the lowest government spending per person per year on health [17] and it is sobering to reflect that these data were collected in Yangon, one of the better resourced regions in the country [18]. Meanwhile, 70% of the population reside in rural areas, where resources and access to skilled health personnel are far more limited [2] and it is these areas—particularly the North and East of the country—where the HIV burden is greatest and programmes for PLHIV less developed [19–22].

The high prevalence of TB in this cohort emphasises the importance of integrating TB and HIV services [23] and brings into sharp relief the very low rates of IPT prescription, although the fact that co-trimoxazole was prescribed commonly demonstrates that there is no reluctance to prescribe prophylaxis per se [24]. Anecdotally, many Myanmar clinicians express concerns about the possibility of IPT leading to the development of resistance or adverse drug reactions, but there is no reason to think that this would occur at a higher rate than in other countries with a high TB prevalence where IPT has shown unequivocal benefit [25, 26]. While the number of patients prescribed IPT in our sample is too small to draw any broad conclusions, it may reassure sceptical Myanmar clinicians that no patients receiving IPT died or required TB treatment during follow-up. In a country where PLHIV are still presenting with advanced immunodeficiency, it is hoped that there is less resistance to the rollout of expanded prophylaxis regimens which have recently been shown to reduce mortality and hospitalisation without increased serious adverse events [27].

The majority of new HIV infections in Myanmar are in PWID (HIV prevalence in this population in 2014: 23.1%), MSM (HIV prevalence in 2014: 6.6%), and FSW (HIV prevalence in 2014: 6.3%) [6], but stigma and discrimination against these populations in Myanmar impose substantial barriers to their access to HIV prevention and care [28]. Both Waibargi and IGH are large hospitals running extremely busy, open-plan outpatient departments. While it appears likely that patients are under-reporting these risk behaviours and/or clinicians are under-documenting them, neither hospital provides an ideal setting for their disclosure. Unfortunately, this is a situation that is replicated around the country; it is estimated that a significant proportion of the KAPs are not being reached by the National AIDS Programme (NAP) and its partners, and an even smaller proportion are being tested for HIV [28].

People who inject drugs are a particularly challenging group to reach, reflected by the fact that this group accounted for 39% of all new infections in 2014 [6]. The fact that the rates of injecting drug use are highest in remote—and sometimes politically unstable—areas in the North of the country adds another layer of complexity to service delivery. However, there are positives: there has been a significant scaling-up of harm reduction activities, including the provision of methadone maintenance therapy and the possession of hypodermic needles is no longer illegal. Meanwhile, supportive drop-in-centres (DICs) continue to be the mainstay of prevention efforts targeting MSM and FSW, providing education, free condoms and outreach programmes. While support groups have been shown to reduce mortality and improve clinical outcomes [29], they undoubtedly improve the quality of life of many MSM and FSW who would otherwise feel ostracised in what remains a conservative country. Some of these DICs also offer onsite health care services and ART access.

The finding that outcomes were worse at Waibargi Hospital—a specialist infectious disease hospital—than IGH—a general hospital—is likely explained by the fact that Waibargi Hospital manages cases that are more complex than IGH. Indeed IGH refers its complicated patients with multiple comorbidities to Waibargi Hospital. Waibargi Hospital also receives more referrals from outside the Yangon region, which hinders optimal follow up care and almost certainly contributes to that hospital’s higher rate of loss to follow up. The association between unemployment—a marker of socioeconomic disadvantage—and death is unsurprising, although the high rate of unemployment in the cohort emphasises the bidirectional relationship between HIV and poverty [30].

There are positives in these data: almost 85% of patients who were prescribed ART had an increase in their CD4+ count and even with high rates of advanced symptomatic disease, overall survival and retention in care was relatively good when compared to other series from low and middle income countries [13, 15, 31, 32]. However, the reality is that Myanmar’s HIV program is grossly underfunded [33]. Total spending for HIV in Myanmar was approximately USD 84.1 million in 2015—an increase from USD 68.9 million in 2014—but this programme is heavily reliant on the Global Fund, and current funding levels are insufficient to reach Myanmar’s stated objectives [34].

One of the greatest issues in the management of HIV in Myanmar is the profoundly under-developed state of laboratory services [35]. The clinical management of other common diseases in Myanmar—such as malaria—requires less laboratory support [36, 37]; however, this is assuredly not the case with HIV. The absence of viral load testing in the public health system precludes early recognition of poor adherence, drug malabsorption and viral resistance, but despite data to support its cost-effectiveness [38], there are enormous challenges in implementing viral load testing in resource-limited settings when even basic laboratory services are lacking [39, 40].

Our study has limitations. Although the data are collected from two of the largest ART prescribing centres in Myanmar’s biggest city, they are not necessarily generalisable to the entire country. There are NGOs that target—and undoubtedly reach—KAPs more effectively and IPT is a key component of the care provided by these services. The data presented here are retrospective and relatively basic; to guide policy in the future, it will be important to collect detailed data prospectively and to link these data with the delivery of services and clinical outcomes. It will be essential to have robust quality assurance mechanisms to ensure that the collected data are complete, timely and accurate if they are to inform optimal policy.

The Myanmar National AIDS Programme (NAP) plans to increase access to public sector ART. It aims to achieve this through further decentralisation of HIV testing and ART prescription, transitioning to a “Test-and-Treat” strategy and expanded co-operation with non-government organisations [6]. There is a particular focus on improving the prevention of mother-to-child transmission, and the integration of TB and HIV services. There is the recognition that the strengthening of laboratory capacity is essential if these ambitious plans are to be to implemented [34]. The NAP acknowledges a failure to reach KAPs and it is hoped that the strengthening of community-based support groups and the integration of harm reduction strategies into community-based settings may improve the delivery of care to these vulnerable populations.

## Conclusions

People living with human immunodeficiency virus at two of the largest ART prescription centres in Myanmar are presenting late in their disease course, increasing their risk of death and disease and the likelihood of viral transmission. Although retention rates and survival were relatively good at these centres, a centralised model of ART prescription is struggling to deliver care to the KAPs that bear the greatest burden of disease in the country. While TB co-infection is very common in Myanmar, IPT is frequently not prescribed despite its proven efficacy. Sustained political and financial commitment, health system strengthening, reliable data collection and flexible, evidence-based health policies are required to build on recent progress in the care of PLHIV in the country.

## Abbreviations

ART: anti-retroviral therapy
DIC: drop in centre
EFV: efavirenz
FSW: female sex workers
FTC: emtricitabine
HIV: human immunodeficiency virus
IGH: Insein General Hospital
IPT: isoniazid prophylaxis therapy
IQR: interquartile range
KAP: key affected populations
MSM: men who have sex with men
NAP: National AIDS Programme
NGO: non-government organisation
PLHIV: people living with human immunodeficiency virus
PWID: people who inject drugs
TB: tuberculosis
TDF: tenofovir disoproxil fumarate
USD: United States dollars
WHO: World Health Organization
